# LINC00707 Regulates miR-382-5p/VEGFA Pathway to Enhance Cervical Cancer Progression

**DOI:** 10.1155/2021/5524632

**Published:** 2021-06-29

**Authors:** Hua Guo, Jing Li, Furong Fan, Ping Zhou

**Affiliations:** ^1^Department of Clinical Laboratory, Hospital of Chengdu University of Traditional Chinese Medicine, Chengdu, China; ^2^Nephrology Department I, Hospital of Chengdu University of Traditional Chinese Medicine, Chengdu, China; ^3^Maternal Health Care Department, Wuhan Children's Hospital (Wuhan Maternal and Child Healthcare Hospital), Tongji Medical College, Huazhong University of Science and Technology, Wuhan, China

## Abstract

Long noncoding RNAs (LncRNAs) are reported to exhibit crucial roles in cancer progression. LINC00707 is recently indicated to be a significant oncogene in various cancers. Up to now, the mechanism of LINC00707 in cervical cancer is still unclear. In this study, our present work was designed to study the biological effects of LINC00707 in cervical cancer. Firstly, the expression level of LINC00707 in cervical cancer was tested. We observed LINC00707 expression was greatly increased in cervical cancer. Then, we assessed the detailed effect of LINC00707 on the development of cervical cancer using CCK-8 assay, Transwell assays, and tumor xenograft experiments. Gain-of-function and loss-of-function assays revealed the function of LINC00707 in cervical cancer progression. In addition, the action of LINC00707 in cervical cancer cells was studied using bioinformatic tools and luciferase reporter experiment. It was displayed that loss of LINC00707 significantly repressed cell growth of cervical cancer. Meanwhile, restoration of LINC00707 expression obviously induced cervical cancer cell growth. Then, we predicted that LINC00707 could serve as a molecular sponge for miR-382-5p to modulate VEGFA expression in cervical cancer. Subsequently, lack of VEGFA expression reversed the influence of miR-382-5p knockdown on cervical cancer cells. In conclusion, our findings evidenced the significant role of LINC00707-miR-382-5p-VEGFA network in cervical cancer and it can provide an attractive target.

## 1. Introduction

Cervical cancer is a second most frequent malignant tumor among women across the world [1]. Recently, many therapeutic methods are developed, such as surgery, radiation oncology, and chemotherapy. However, the 5-year survival rate for cervical patients still remains unsatisfactory [2, 3]. Therefore, further revealing critical molecular mechanisms driving cervical cancer progression and developing more effective therapies for cervical cancer are urgently needed.

LncRNAs belong to noncoding RNA family, with over 200 nucleotides, which lack protein-coding capacity [4–6]. LncRNAs can modulate numerous cellular biological processes including apoptosis, proliferation, and differentiation [7, 8]. LncRNAs can exhibit crucial roles in various cancers [9]. MicroRNAs are able to target the 3'UTR of their targeting mRNA, which leads to translational inhibition [10, 11]. Recent studies report that LncRNAs serve as microRNA “sponges” to inhibit the activity of microRNAs [12]. For instance, B3GALT5-AS1 is able to repress colon cancer progression via sponging miR-203 [13]. LINC00511 promotes to breast cancer development through regulating miR-185-3p/E2F1/Nanog [14]. In cervical cancer, XLOC_006390 can facilitate tumor development by sponging miR-331-3p and miR-338-3p [15].

LINC00707 is located on chromosome 10p14 involved in many cancers. For instance, LINC00707 can promote breast cancer cell growth via modulating miR-30c and CTHRC1 [16]. Loss of LINC00707 can enhance cisplatin sensitivity in lung cancer cells through sponging miR-145 [17]. In addition, LINC00707 can accelerate clear cell renal cell carcinoma development [18]. Nevertheless, the underlying mechanism of LINC00707 in cervical cancer is largely uninvestigated. Therefore, identifying the function of LINC00707 during cervical cancer is beneficial for appropriate therapy of cervical cancer development.

Here, we studied the biological role of LINC00707 in cervical cancer. We found LINC00707 was elevated in cervical cancer while miR-382-5p was decreased. LINC00707 could negatively modulate miR-382-5p expression in vitro. Additionally, VEGFA was predicted as a target for miR-382-5p. Thus, we hypothesized LINC00707 functioned as a novel biomarker in cervical cancer via modulating miR-382-5p and VEGFA.

## 2. Materials and Methods

### 2.1. Clinical Samples

20 cervical cancer patients undergoing a mastectomy at Hospital of Chengdu University of Traditional Chinese Medicine were recruited to our research. All the specimens were pathologically confirmed as cervical cancer resection. Tumor and adjacent tissues were maintained in liquid nitrogen for future experiment. The study was approved by the Ethics Committee of Hospital of Chengdu University of Traditional Chinese Medicine. Written informed consents were acquired from cervical cancer patients.

### 2.2. Cell Culture

HT-3, HCC94, CaSki, C-33A, MS751, and HEKn cells were obtained from ATCC (Manassas, VA, USA). Then, DMEM medium added with 10% FBS and 100 U/mL Penicillin/Streptomycin was used to incubate all the cells in a 5% CO_2_ incubator. Cells were collected at 90% confluence, and the medium was changed every 48 h.

### 2.3. Cell Transfection

Cells at exponential stage were utilized to do transfection. miR-382-5p mimics, inhibitors, LINC00707 siRNA, VEGFA shRNA, and the corresponding negative controls were obtained from GenePharma (Shanghai, China). Lipofectamine 3000 reagents were used to transfect the vectors and microRNAs into cervical cancer cells. Full-length complementary DNA (cDNA) of human LINC00707 was amplified from the messenger RNA (mRNA) of cervical cancer cells. Overexpression of LINC00707 and loss of LINC00707 were carried out by infection with LV-LINC00707 and LV-shLINC00707, respectively. The LINC00707 sequence was amplified and inserted into the vector to overexpress LINC00707 with a blank plasmid vector as an NC.

LINC00707 siRNA-01: 5′-GGCUUUCCAUGACCCAUAATT-3′, siRNA-02: 5′-GCAGGAACAUCACCAUCUUTT-3′, siRNA-03: 5′-GGAAGCCACUCCUGCAUUUTT-3′, and si-NC: 5′-UUCUCCGAACGUGUCACGUTT-3′. The sequence for generating VEGF shRNA is as follows: AAATGTGAATGCAGACCAAAGTTCAAGAGACTTTGGTCTGCATTCACATTT. The sequence for generating LINC00707 shRNA is as follows: GCACTGGATCAATTCCAATTA.

### 2.4. CCK-8 Assay

CCK-8 assay (Dojindo, Shanghai, China) was employed to test cell proliferation. 10 *μ*L CCK-8 reagent was added for 2 h. Then, dual-wavelength microplate reader was employed to test wavelength of 450-490 nm.

### 2.5. EdU Assay

To test cell proliferation, 50 nmol/L EdU (RiboBio, Guangzhou, China) was utilized. Cells were indicated with 1 mL Cell-LightTM EdU Apollo®488. Then, DAPI was utilized to stain the nucleus for 30 min. Fluorescence intensity was assessed under an inverted microscope.

### 2.6. Transwell Assays

To do migration assay, cells were grown into the upper chamber of Transwell assay inserts added with 200 *μ*l serum-free DMEM with an 8 mm pores-membrane. Cells on surface were stained using crystal violet. Afterwards, cells were photographed using a digital microscope. To do invasion assay, cells were plated in the top chamber with a Matrigel-coated membrane. 48 h later, the invasive ability was evaluated using the migration assay as above.

### 2.7. TUNEL

TUNEL assay was used to test the induction of apoptosis using In Situ Cell Death Detection Kit. In brief, after cells were pretreated using 10 nmol/L DTX, cells in each sample were stained under the manufacturer's instructions. Then, an EVOS FL microscope was utilized to count the positively stained cells.

### 2.8. Flow Cytometry Analysis

After cells were digested using trypsin, cells were indicated with PE-conjugated anti-Ki-67 monoclonal antibodies at 4°C. Then, the ratio of positive tumor cells was calculated using detection of fluorescence intensity on FACS Calibur (Becton-Dickinson, CA, USA).

### 2.9. Western Blot Assay

20 *μ*g proteins was carried out on SDS-PAGE electrophoresis. PVDF membranes were blocked by 5% skimmed dry milk. Rabbit polyclonal to VEGFA and mouse monoclonal to GAPDH (Cell Signaling Technology, Danvers, MA, USA) were employed as the primary antibodies. After incubated at 4°C, TBST was used to wash the membranes. Next day, the membranes were continuously incubated with secondary antibodies. Then, the immunoblots were visualized using enhanced chemiluminescence reagent.

### 2.10. qRT-PCR

RNA was extracted using TRIzol reagents, and isolated RNA was transcribed into cDNA using TaKaRa Reverse Kit (TaKaRa, Dalian, China). Afterwards, real-time PCR was performed on StepOne Real-Time PCR System and calculated using the 2^−∆∆Ct^ method. Sequences of the primers were provided in Table 1.

### 2.11. Dual-Luciferase Reporter Analysis

Dual Luciferase Reporter was used to test the correlation between LINC00707, miR-382-5p, and VEGFA. LINC00707 (or VEGFA 3′UTR) fragments with the predicted WT or MUT miR-382-5p binding sites were cloned into the vector pmirGLO (Promega, Madison, WI, USA). To carry out the luciferase activity assay, 1 × 10^4^ cells were cotransfected with 50 nM pmirGLO-LINC00707-WT (or pmirGLO-VEGFA-WT) or pmirGLO-LINC00707-MUT (or pmirGLO-VEGFA-MUT) and 50 nM miR-382-5p mimics, inhibitors, or miR-NC. Dual Luciferase Reporter Assay system was utilized to measure the luciferase activity.

### 2.12. RIP Analysis

RIP was conducted using a Magna RIP RNA-Binding Protein Immunoprecipitation kit (Millipore, Bedford, MA, USA). Cell lysates were indicated with magnetic beads conjugated with negative control normal mouse IgG or human anti-Ago2 antibody (Millipore, Bedford, MA, USA). Immunoprecipitated RNA was detected by qRT-PCR analysis.

### 2.13. Tumor Xenograft

Five-week-old specific pathogen free mice were obtained from Beijing HFK Bio-Technology (Beijing, China). C-33A was transfected with LV-shLINC00707 or LV-LINC00707, and then, 5 × 10^6^ transfected cells were injected to each mouse in situ in 200 *μ*l PBS. After 42 days, mice were euthanized by CO_2_ inhalation. Our research was under the Guide for the Care and Use of Laboratory Animals of the NIH.

### 2.14. Immunohistochemical (IHC) Analysis

Briefly, paraffin-embedded tissues were cut into 3 *μ*m sections and then dewaxed with xylene and rehydrated in graded ethanol. Antigen retrieval was carried out using citrate buffer, pH 6.0. Sections were heated at 97°C for 20 min for antigen retrieval. Following a brief proteolytic digestion and peroxidase blocking, the sections were incubated with a Ki-67 polyclonal antibody at 4°C overnight. Next day, HRP/Fab polymer conjugate was used as the secondary antibody. Finally, the sections were stained by diaminobenzidine substrate and counterstained using hematoxylin.

### 2.15. Statistical Analysis

Statistical analysis was performed using the GraphPad Prism 6.0 software. Student's *t*-test was employed to assess the data between two groups. The comparison among multiple groups was made using one-way ANOVA. Significant level was considered as *P* value <0.05.

## 3. Results

### 3.1. Downregulation of LINC00707 Restrained Cervical Cell Growth In Vitro

**Figure 1:**
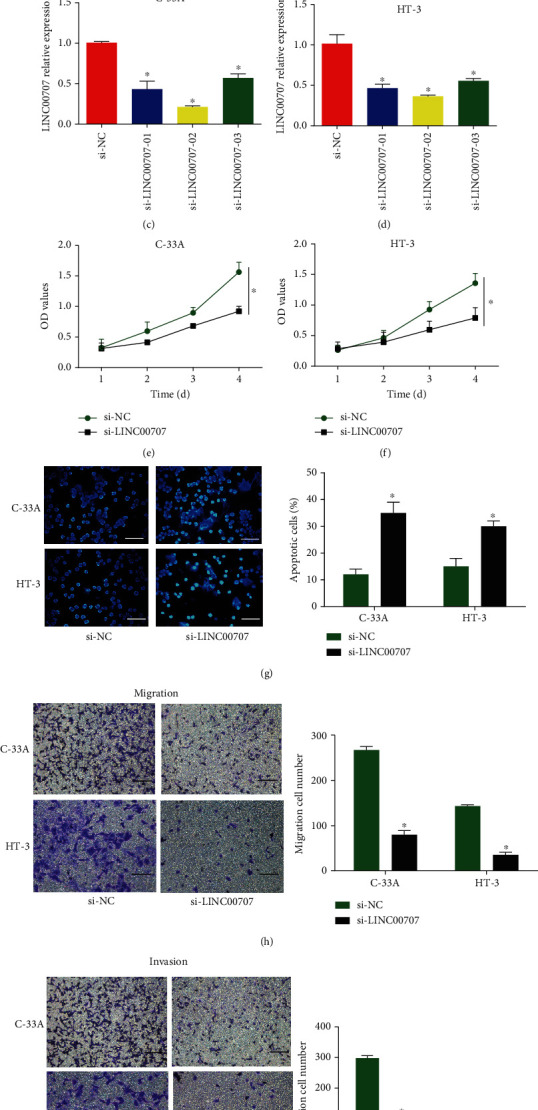
LINC00707 was increased in cervical cancer. (a) RT-PCR indicated the LINC00707 expression in cervical cancer tissues (*n* = 20) and adjacent normal tissues (*n* = 20). (b) LINC00707 expression in cervical cancer cells (HT-3, HCC94, CaSki, C-33A, MS751, and normal cervical cell HEKn cells). (c, d) The expression level of LINC00707 in C-33A and HT-3 cells was tested by RT-PCR. (e, f) Cell proliferation was tested after transfection with LINC00707 siRNA using CCK-8 assay. (g) Cell apoptosis was evaluated using TUNEL assay (scale bar = 50 *μ*m). (h, i) Transwell migration and invasion assay were performed. ^∗^*P* < 0.05 and ^∗∗∗^*P* < 0.001.

At first, data indicated that LINC00707 was strongly elevated in cervical cancer specimens (Figure 1(a)). LINC00707 expression was obviously upregulated in cervical cancer cells (HCC94, CaSki, MS751, HT-3, and C-33A) in comparison to human epidermal cells (HEKn) (Figure 1(b)). Moreover, HT-3 and C-33A cells were chosen to carry out subsequent assays. Next, we focused on the effect of LINC00707 on biological function of cervical cancer cells. In Figures 1(c) and 1(d), expression of LINC00707 was reduced after transfection of three specific siRNAs. Among them, si-LINC00707-02 exhibited the best interference efficiency. CCK-8 results indicated the viability ability of cervical cancer cells was repressed by LINC00707 knockdown (Figures 1(e) and 1(f)). Then, TUNEL analysis proved that cervical cancer cell apoptosis was markedly boosted by LINC00707 siRNA (Figure 1(g)). Transwell assay revealed that migration and invasion number of HT-3 and C-33A cells were repressed due to silencing of LINC00707 in Figures 1(h) and 1(i). To summarize, LINC00707 silencing restrained cervical cancer cell progression.

### 3.2. Upregulation of LINC00707 Induced Cervical Cancer Cell Growth

Moreover, C-33A and HT-3 cells were infected with LV-LINC00707. As shown in Figures 2(a) and 2(b), LINC00707 was greatly increased by LV-LINC00707. In Figure 2(c), Ki-67 positive cell ratio was obviously induced by the overexpression of LINC00707. In addition, as demonstrated in Figures 2(d) and 2(e), C-33A and HT-3 cell migration and invasion were significantly enhanced by LV-LINC00707. These manifested increased LINC00707 promoted cervical cancer cell proliferation, migration, and invasion.

### 3.3. miR-382-5p Was Decreased in Cervical Cancer and Negatively Modulated by LINC00707

**Figure 3:**
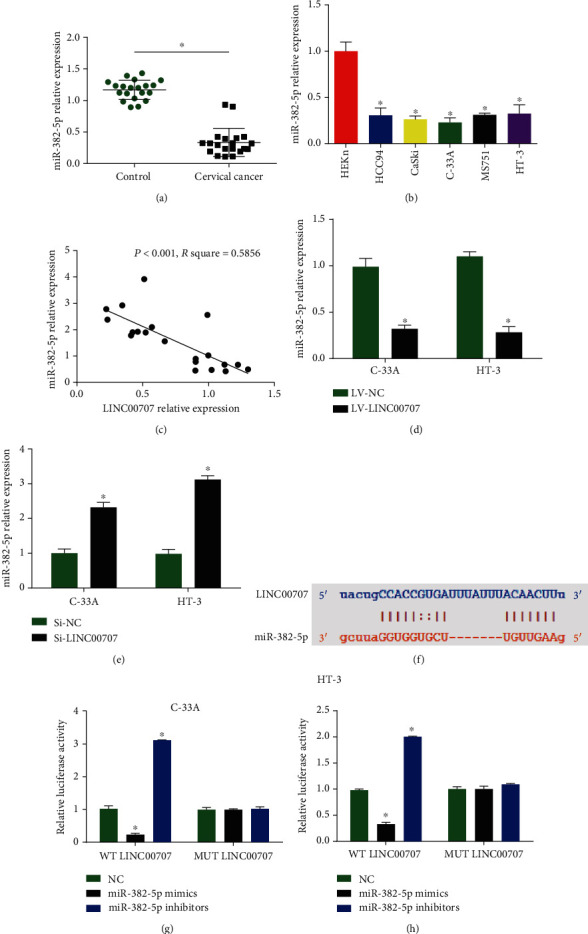
LINC00707 sponged miR-382-5p. (a) RT-PCR indicated miR-382-5p expression in cervical cancer. (b) miR-382-5p expression in cervical cancer cells (HT-3, HCC94, CaSki, C-33A, MS751, and HEKn cells). (c) Pearson analysis of LINC00707 and miR-382-5p in cervical cancer tissues. (d, e) miR-382-5p expression in C-33A and HT-3 cells infected with LV-LINC00707 or LINC00707 siRNA. (f) Binding sites between LINC00707 and miR-382-5p. (g, h) Luciferase activity was evaluated in C-33A and HT-3 cells cotransfected with LINC00707-WT or LINC00707-MUT reporter and miR-382-5p inhibitors or mimics. ^∗^*P* < 0.05.

Then, to explore the molecular mechanism of LINC00707, the potential miRNAs were evaluated and miR-382-5p was predicted as the target using http://starbase.sysu.edu.cn/. In cervical cancer tissues and cells, miR-382-5p was reduced as shown in Figures 3(a) and 3(b). In addition, in Figure 3(c), a positive correlation between LINC00707 and miR-382-5p was indicated. Knockdown of LINC00707 could promote miR-382-5p expression, and overexpression of LINC00707 reduced miR-382-5p expression (Figures 3(d) and 3(e)). In Figure 3(f), we first constructed LINC00707-WT and LINC00707-MUT luciferase reporter plasmids. miR-382-5p strikingly reduced the luciferase activity of LINC00707-WT while inhibitors of miR-382-5p enhanced the luciferase activity in Figures 3(g) and 3(h). These indicated miR-382-5p was negatively modulated by LINC00707.

### 3.4. miR-382-5p Targeted VEGFA

**Figure 4:**
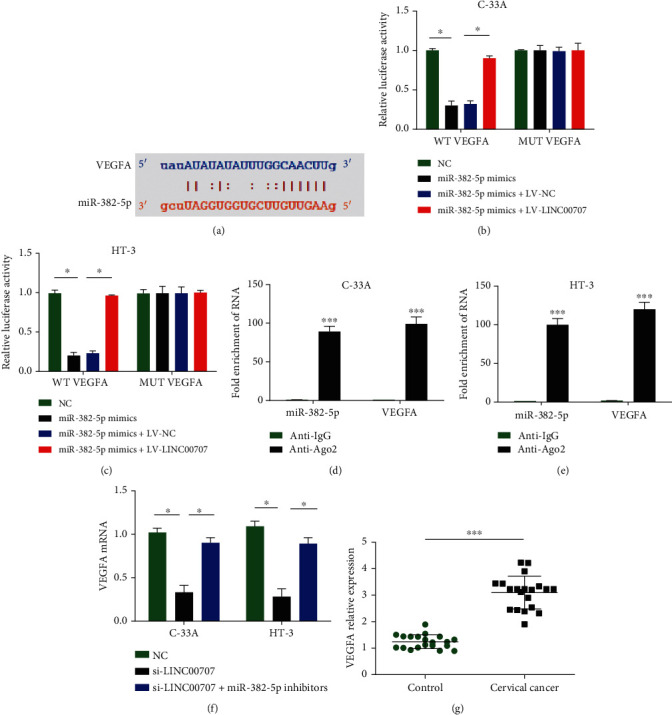
VEGFA was a target for miR-382-5p. (a) Binding regions between VEGFA and miR-382-5p. (b, c) Luciferase activity was evaluated in C-33A and HT-3 cells cotransfected with VEGFA-WT or VEGFA-MUT reporter and miR-382-5p mimics or LV-LINC00707. (d, e) RIP experiment was carried out to assess the interplay between VEGFA and miR-382-5p. (f) VEGFA mRNA expression in C-33A and HT-3 cells. (g) VEGFA mRNA expression in cervical cancer tissues. ^∗^*P* < 0.05 and ^∗∗∗^*P* < 0.001.

Furthermore, VEGFA was predicted as a target for miR-382-5p in Figure 4(a) using http://starbase.sysu.edu.cn/. Then, we explored the feasible relationship between miR-382-5p and VEGFA in cervical cancer. As demonstrated in Figures 4(b) and 4(c), miR-382-5p mimics apparently inhibited the luciferase activity of VEGFA-WT, which was countervailed through adding LV-LINC00707. The luciferase activity of VEGFA-MUT was not affected by either miR-382-5p mimics or LV-LINC00707 alone and in combination. VEGFA and miR-382-5p presented high enrichment in Ago2 group compared to IgG group, which indicated the fold enrichment of VEGFA and miR-382-5p in RISC (Figures 4(d) and 4(e)). In Figure 4(f), it was displayed that VEGFA mRNA was reduced by loss of LINC00707, which was reversed by lack of miR-382-5p. In addition, in Figure 4(g), VEGFA mRNA was significantly increased in cervical cancer tissues. These manifested that VEGFA was a downstream target of miR-382-5p.

### 3.5. miR-382-5p Inhibited Cervical Carcinoma Cell Growth via Targeting VEGFA

**Figure 5:**
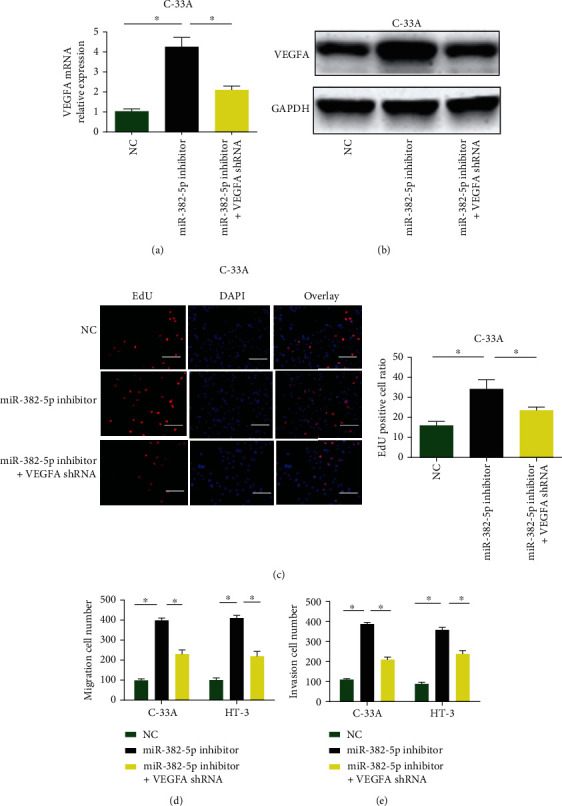
Loss of VEGFA repressed cervical cancer cell growth. (a, b) VEGFA expression in C-33A and HT-3 cells transfected with miR-382-5p inhibitors and VEGFA shRNA. (c) EdU assay was used to evaluate cell proliferation (scale bar = 50 *μ*m). (d, e) Cell migration and invasion. ^∗^*P* < 0.05.

To investigate whether miR-382-5p influenced the biological function of CC cells through targeting VEGFA, we performed rescue assays. C-33A cells were transfected with miR-382-5p inhibitors and VEGFA shRNA. VEGFA mRNA and protein level was inhibited by miR-382-5p inhibitor, which was reversed by VEGFA shRNA (Figures 5(a) and 5(b)). EdU assay indicated C-33A cell proliferation was induced by loss of miR-382-5p while loss of VEGFA decreased cell proliferation (Figure 5(c)). Additionally, C-33A cell migration and invasion capacity was markedly reduced by silencing VEGFA in vitro. These displayed miR-382-5p inhibited cervical cancer cell growth via targeting VEGFA.

### 3.6. LINC00707 Contributed to Cervical Cancer Growth In Vivo through Modulating miR-382-5p and VEGFA

**Figure 6:**
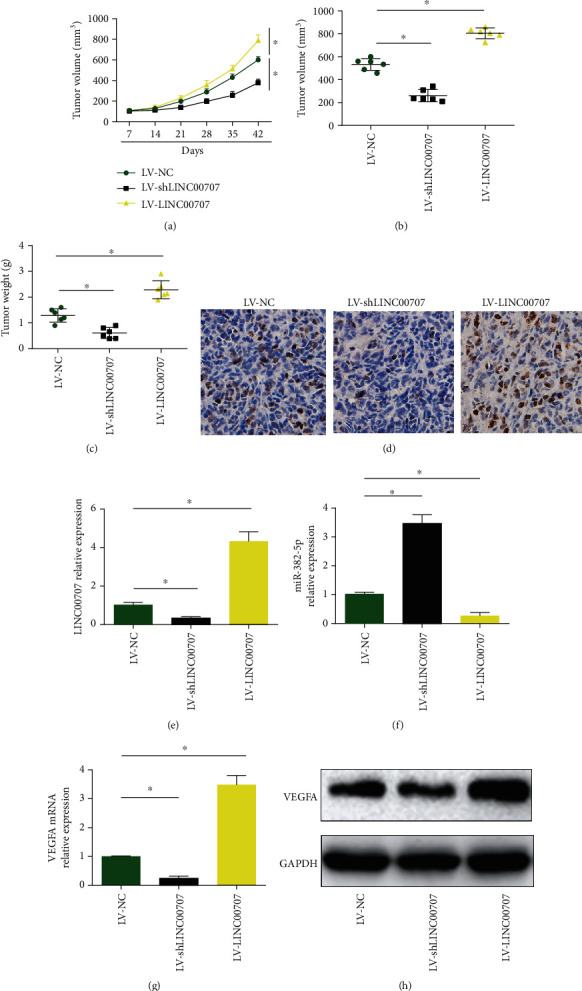
Loss of LINC00707 restrained cervical cancer growth through sponging miR-382-5p in vivo. BALB/c nude mice were injected with C-33A cells infected with LV-NC (six mice) or LV-LINC00707 (six mice) or LV-shLINC00707 (six mice). (a) Tumor volume in a time-dependent manner. (b) Tumor weight when the mice were sacrificed. (c) Tumor volume when the mice were sacrificed. (d) IHC analysis of Ki-67 (scale bar = 50 *μ*m). (e) Expression of LINC00707. (f) Expression of miR-382-5p. (g, h) Expression of VEGFA. ^∗^*P* < 0.05.

Furthermore, eighteen nude mice were injected with C-33A cells infected with LV-NC (six mice) or LV-LINC00707 or LV-shLINC00707 (six mice). Tumor tissues were peeled from the mice, and the tumor weight and volume were evaluated. Then, tumor volume was increased by overexpressed LINC00707 while reduced by knockdown of LINC00707 time dependently (Figure 6(a)). In Figures 6(b) and 6(c), we found that tumor volume and tumor weight were reduced by the loss of LINC00707 while elevated by LINC00707 overexpression. In addition, Ki-67 staining analysis was exhibited in Figure 6(d), and we observed that overexpression of LINC00707 group displayed strong Ki-67 staining tumor tissues. We confirmed that expression of LINC00707 in the tumor tissues was repressed by LV-shLINC00707 and induced by LV-LINC00707 in Figure 6(e). miR-382-5p/VEGFA axis participated in LINC00707-mediated cervical cancer progression (Figures 6(f)–6(h)).

## 4. Discussion

LncRNAs are reported to modulate various physiological processes [19, 20]. Increasing researches have indicated that many LncRNAs are closely associated with many cancers [21]. In this research, we identified LINC00707 was elevated in cervical cancer tissues and cells. Loss of LINC00707 greatly inhibited the tumor growth of cervical cancer. In addition, the LINC00707 abnormal high expression contributed to cervical cancer growth. Besides, miR-382-5p was predicted as a target for LINC00707, and VEGFA was a target for miR-382-5p. Our findings indicated that LINC00707 and miR-382-5p could be used as novel markers of cervical cancer and were potential therapeutic targets for cervical cancer treatment via modulating VEGFA.

Recent studies display LncRNAs are related with cervical cancer. XIST can accelerate cervical cancer via inducing Fus by sponging miR-200a [22]. TCONS_00026907 contributes to cervical cancer through sponging miR-143-5p [23]. In our study, we displayed that LINC00707 increased in cervical cancer tissues and cells. Our study also disclosed that LINC00707 acted as a crucial regulator in cervical cancer development and facilitated cervical cancer cell propagation and promoted tumor growth in vitro and in vivo.

miR-382-5p was predicted as a target for LINC00707. miR-382-5p level is decreased in various cancers. CircRNA-UBAP2 can promote the proliferation and restrain ovarian cancer apoptosis via regulating miR-382-5p and PRPF8 [24]. miR-382-5p can aggravate breast cancer development through regulating RERG/Ras/ERK signaling [25]. We reported a decreased expression of miR-382-5p in cervical cancer. Our data displayed that miR-382-5p was negatively regulated by LINC00707. Collectively with the other report, our data highlighted the functional characteristics of miR-382-5p as a tumor inhibitor. Furthermore, we demonstrated that LINC00707 functioned as a competing endogenous RNA (ceRNA) to affect miR-382-5p activity and regulate the miR-382-5p target gene VEGFA.

VEGFA is upregulated in a lot of cancers [26]. It has been well documented that VEGF is closely associated with cancers [27, 28]. miR-144 can restrain cervical cancer growth and metastasis through targeting VEGFA [29]. miR-203 can inhibit cervical cancer growth and angiogenesis through targeting VEGFA [30]. Here, we used a luciferase reporter assay and demonstrated VEGFA was a direct target of miR-382-5p. VEGFA downregulation could rescue the effect caused by miR-382-5p inhibitors. We could conclude LINC00707 induced cervical cancer development via regulating miR-382-5p and VEGFA. Furthermore, as we displayed, cervical cancer cell apoptosis, proliferation, migration, and invasion were all impacted by VEGFA. Downstream targets of VEGFA should be explored. Hallmark responses of VEGFA involve a number of effectors, such as ERKs and PI3K/Akt [31]. It has been reported that VEGFA expression can activate PI3K/Akt signaling [32]. Therefore, alterations in PI3K signaling pathway should be investigated.

However, there are also some limitations in this study. First, the limited tissue samples were not fully substantiating the accuracy of the results. Second, detailed investigation of genes that comprise the LINC00707/miR-382-5p/VEGFA axis should be further explored. The relationship between LINC00707 and other potential targeting miRNAs needed more researches. The relationship between miR-382-5p and other potential targeting mRNAs needed more attention. In addition, we just focused on two cervical cancer cell lines, which made the conclusion not sufficient enough.

In conclusion, we confirmed that LINC00707 functioned as an oncogene in cervical cancer through forming the LINC00707/miR-382-5p/VEGFA axis. These data could provide a novel biomarker and insight for ceRNA mechanism in cervical cancer progression.

## Figures and Tables

**Figure 2:**
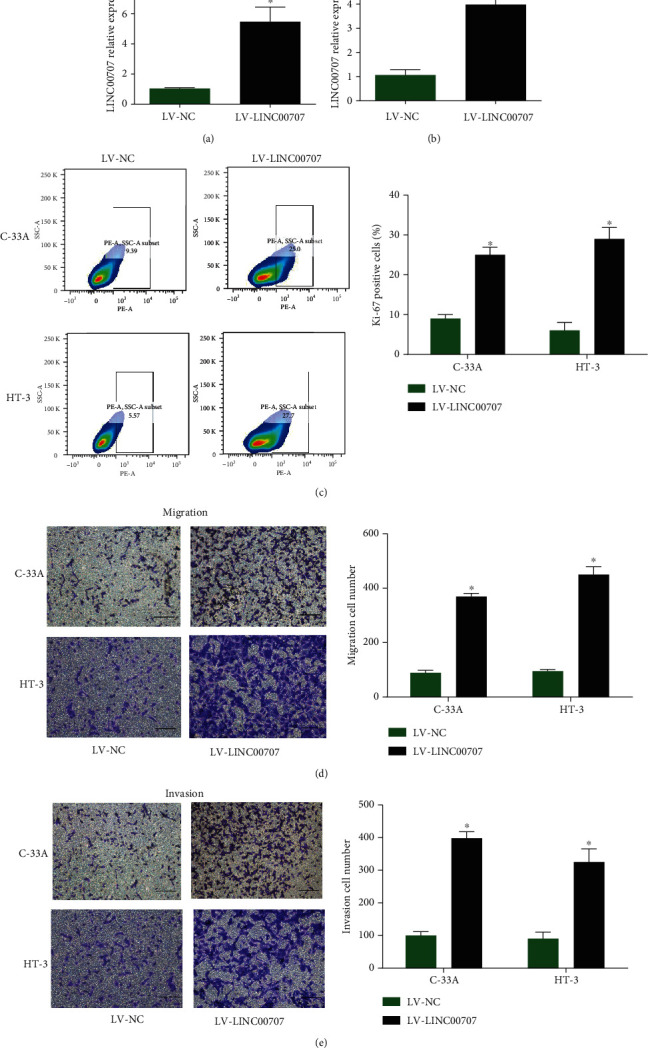
LINC00707 induced cervical cancer cell growth in vitro. (a, b) LINC00707 expression in C-33A and HT-3 cells infected with LV-LINC00707 was assessed by RT-qPCR assay. (c) Ki-67 positive cell ratio. (d, e) Cell migration and invasion (scale bar = 50 *μ*m). ^∗^*P* < 0.05.

**Table 1:** Primers used for real-time PCR.

Genes	Forward (5′-3′)	Reverse (5′-3′)
GAPDH	GCACCGTCAAGGCTGAGAAC	TGGTG AAGACGCCAGTGGA
LINC00707	CCAACAGGGTATCAGAATTCTC	TGCTGACAATAGCCATTAGG
miR-382-5p	CTCGCTTCGGCAGCACA	TATGGTTGTAGAGGACTCCTTGAC
U6	AACGCTTCACGAATTTGCGT	CTCGCTTCGGCAGCACA
VEGFA	CACCGAAGGAGACAGTGAATCC	GCTGTTCTGGAGTAAGCTTGTGC

## Data Availability

The data used to support the findings of this study are included within the article.
